# Meiotic Chromosomal Abnormality Detected in a Heterozygote of *Elymus nutans*

**DOI:** 10.3389/fpls.2022.895437

**Published:** 2022-05-03

**Authors:** Bo Liu, Xiaoyan Tao, Quanwen Dou

**Affiliations:** ^1^Key Laboratory of Adaptation and Evolution of Plateau Biota, Northwest Institute of Plateau Biology, Chinese Academy of Sciences, Xining, China; ^2^University of Chinese Academy of Sciences, Beijing, China; ^3^Qinghai Province Key Laboratory of Crop Molecular Breeding, Northwest Institute of Plateau Biology, Chinese Academy of Sciences, Xining, China

**Keywords:** *E. nutans*, meiosis, fluorescence *in situ* hybridization, deletion, inversion

## Abstract

*Elymus nutans* is an allopolyploid with a genome constitution of StStYYHH (2*n* = 6*x* = 42). Highly frequent intergenomic translocations and chromosomal variations with repeat amplification and deletions in *E. nutans* have been identified in the previous studies. However, more complicated structural variations such as chromosomal inversions or intra-genomic translocations are still unknown in this species, so does the reason for the origin of the chromosomal variations. Heterozygotes with rearranged chromosomes always present irregular meiosis behaviors, which subsequently cause the secondary chromosome rearrangements. Investigation on the meiosis of heterozygotes, especially on the individual chromosome level, may provide the important clues to identify the more complicated chromosome structural variations in the populations, and clarify the origin of the chromosome variations. In this study, meiotic analysis was conducted on a heterozygote plant of *Elymus nutans*, which showed high intra- and inter-genome chromosomal variations, by sequential fluorescence *in situ* hybridization (FISH) and genomic *in situ* hybridization (GISH), with each chromosome clearly recognized. The results showed chromosomal abnormalities at every meiotic stage and abnormalities in frequency variations between different sub-genomes and different individual chromosomes. The abnormalities were revealed as univalent, fragment, rod, or Y shape bivalent in diakinesis; univalent and rod bivalent in metaphase I; lagged and segregated chromatid, bridge, fragment of the sister chromatid, fragment, bridge accompanied with fragment, and unequal segregated chromosome in anaphase I; bridge and lagged chromatid in ana-telophase II; and micronucleus at uninucleate stage. Generally, the St and H genomes harbor more abnormalities than the Y genome. Moreover, a paracentric inversion in 2St was exclusively determined, and another paracentric inversion in 6Y was tentatively identified. In addition, novel deletions were clearly detected in 3H, 4H, 1Y, and 3Y homologous chromosomes; in particular, *de novo* pericentric inversion in 3H was repeatedly identified in metaphase I. The study revealed the chromosomal inversions pre-existed in parents or populations, as well as *de novo* inversions and deletions originated in the meiosis of the heterozygote in *E. nutans*. Moreover, it indicated wide range of meiosis abnormalities on different stages and different chromosomes, and suggests that secondary rearrangements contribute much to the chromosome variations in *E. nutans*.

## Introduction

Chromosomal structural variations, particularly translocation and inversion, play important roles in karyotype evolution, adaptation divergence, and speciation (Dobzhansky and Sturtevant, 1938; Rieseberg, 2001; Todesco et al., 2020). In heterozygotes with rearranged chromosomes, recombination between rearranged regions produces chromatin with duplicated and deficient genes. During the first meiotic division, disjunction patterns can cause varying degrees of sterility in the gametophytic and sporophytic phases. Because viable recombinants from inversion heterozygotes are extremely rare, inversions have been classically defined as recombination suppressors. Gene flow between rearranged regions is greatly restricted by recombination suppression (Livingstone and Rieseberg, 2004). Adaptive genes can be held together in chromosomal regions with structural variations *via* recombination suppression (Rieseberg, 2001; Livingstone and Rieseberg, 2004). Recombination suppression is typically involved in both inversion and translocation (Davisson and Akeson, 1993; Jiang et al., 2007; Dumas et al., 2015; Todesco et al., 2020). In addition, novel variations can be induced by altering the gene expression pattern and producing new chimeric genes near chromosome breakpoints (Lister et al., 1993; Hewitt et al., 2014).

*Elymus nutans* Griseb. is a well-known perennial and caespitose grass belonging to the genus *Elymus* L. in the Triticeae tribe of the family Poaceae, which comprises approximately 150 perennial and exclusively polyploid species (Löve, 1984). *E. nutans* is widely distributed in Central and Eastern Asia and the Himalayas (Bor, 1960; Lu, 1993; Chen and Zhu, 2006). *E. nutans* is an allopolyploid with a genome constitution of StStHHYY (2*n* = 6*x* = 42). The St genome originates from the genus *Pseudoroegneria*, the H genome from *Hordeum*, and the Y genome’s origin is unknown and still on debating (Wang et al., 1994; Lei et al., 2022; Liu et al., 2022). *E. nutans* distributed on the Qinghai-Tibet Plateau show highly divergent adaptation, growing on grassland, bushland, wetland, mountain slopes, and swales, at altitudes from 1,000 to 5,000 m, and is tolerant to various environmental stresses associated with high altitudes, such as cold and drought. *E. nutans* shows great variability in the Qinghai-Tibet Plateau, in morphological characteristics (Zhang et al., 2009), inter-simple sequence repeat analysis (Chen S. Y. et al., 2009), sequence-related amplified polymorphism (Chen Z. H. et al., 2009), amplified fragment length polymorphism (Yan et al., 2010), gliadin variation (Miao et al., 2011), and simple sequence repeats analysis (Chen et al., 2013). In addition, *E. nutans* in this region showed high chromosomal variation. Chromosomal rearrangements (translocations or inversions) were first suggested from regular analysis of chromosome pairing in a hybrid between parents from two different populations (Lu, 1993). Later, 42 chromosomes of *E. nutans* were well recognized and allocated three different genomes (St, H, and Y) by using fluorescence *in situ* hybridization (FISH) with tandem repetitive sequences and total genomic DNA as FISH probes (Dou et al., 2009, 2017). High molecular karyotype variations were identified in intra- and inter-populations of *E. nutans*, and highly frequent intergenomic translocations were identified, and chromosomal variations with repeat amplification and deletions were exclusively identified in the aforementioned studies (Dou et al., 2009, 2017). However, chromosomal inversions are still difficult to determine because of technical limitations. Moreover, the rise in chromosomal variation in *E. nutans* is still unknown.

Meiosis is a unique process in which four haploid daughter cells are generated from a diploid parent cell after a single round of DNA replication and two consecutive nuclear divisions. Accurate segregation requires coordinated execution of conserved processes occurring throughout the two meiotic cell divisions. Meiotic recombination is closely connected to homologous chromosome pairing, crossovers, and reciprocal chromosome segment exchange forms. Recombination during meiosis is initiated by forming double-strand breaks (DSBs) in chromosomal DNA (Zamariola et al., 2014). Misrepair of DNA DSBs, particularly by non-homologous end-joining DSBs, may lead to primary chromosomal structural variations (Schubert, 2007; Gaeta and Chris Pires, 2010). In heterozygotes with rearranged chromosomes, chromosomal abnormalities can occur in different meiosis stages, which can cause variations in the second chromosome structure (Livingstone and Rieseberg, 2004; Schubert, 2007). Low to high frequencies of heterozygotes have been revealed in domesticated and wild populations of *E. nutans* by molecular karyotyping (Dou et al., 2009, 2017). The association between karyotype heterozygosity and low seed fertility has also been demonstrated, reflecting the involvement of chromosome rearrangements in the heterozygotes of *E. nutans* (Liu et al., 2021). In this study, an individual of *E. nutans* with a heterozygous karyotype and low fertility was used, and detailed cytological observations were carried out at different meiosis stages with each chromosome clearly identified by FISH. The results are expected to reveal the particular inversions previously undiscovered and help to elucidate the rise of chromosome variation in *E. nutans*.

## Materials and Methods

### Plant Materials

One *E. nutans* plant (A02-1) showed a low seed fertility rate (lower than 10%) and was identified in the field near Qinghai Lake (Qinghai, China) at an altitude of approximately 3,200 m. Owing to its perennial nature, the vegetative mass of the original plant (A02-1) was singly transplanted to an experimental plot in Xining, Qinghai, at an altitude of approximately 2,200 m for further investigation.

### Mitotic Chromosome Preparation

Secondary roots of the A02-1 were collected at lengths of 1–2 cm, pretreated with N_2_O at 7 atm for 2 h, and fixed in 3:1 (v/v) ethanol:glacial acetic acid. Then, chromosome spread and slide preparation were performed as described by Xie et al. (2020).

### Meiotic Chromosome Preparation

Inflorescences were collected during the early flowering stage, morphologically at a distance of 1–2 cm between the flag leaf and the next leaf. The collected inflorescences were fixed in Carnoy II solution (ethanol:glacial acetic acid:chloroform = 6:1:3) for 24 h; then stored in 70% alcohol at −20^°^C until use. First, the meiotic cells in pollen mother cells were examined using a phase-contrast microscope after squashing in 45% acetic acid. Further processing followed the mitotic chromosome preparation. More than 50 cells in each invested stage were arrested and analyzed.

### Fluorescence *in situ* Hybridization and Genomic *in situ* Hybridization

Four repetitive sequences were used as markers for chromosomal discrimination: satellite DNA pAs1 (Rayburn and Gill, 1986) and pSc119.2 (Bedbrook et al., 1980), 45S rDNA (Gerlach and Bedbrook, 1979), and microsatellite (AAG)_10_, which are ideal chromosome markers widely used in Triticeae species (Pedersen and Langridge, 1997; Cuadrado and Schwarzacher, 1998; Cuadrado and Jouve, 2002). Designated oligonucleotides pAs1-1 plus pAs1-2 represent pAs1, Oligo-pTa71-2 represents 45S rDNA, Oligo-pSc119.2-1 plus Oligo-pSc119.2-2 represent pSc119.2 (Tang et al., 2014), respectively. Repetitive sequences pSc119.2 and (AAG)_10_ were end-labeled using fluorescein amidite (FAM; green), and 45S rDNA and pAs1 were end-labeled using carboxy-tetramethylrhodamine (TAMRA; red) (Sangon Biotech Co., Ltd., Shanghai, China) to generate FISH probes. Genomic DNA from *Hordeum bogdanii* Wilensky (2*n* = 2*x* = 14, HH), *Pseudoroegneria stipifolia* (Czern. ex Nevski) Á. Löve (2*n* = 2*x* = 14, StSt) was labeled with fluorescein-12-dUTP and tetramethyl-rhodamine-5-dUTP, respectively, using the random primer labeling method described by Dou et al. (2009). FISH and GISH were performed as previously described by Xie et al. (2020). Images were obtained using fluorescence microscopy (BX63; Olympus, Tokyo, Japan). Karyotyping of the mitotic cell chromosomes was referring to the molecular karyotypes described by Dou et al. (2017), and chromosomes were arranged as 1–7 according to decreasing values of arm ratio and the relative length.

### Statistics

The number of chromosomal abnormalities at every meiotic stage in each pollen mother cell was counted and averaged to represent the abnormality frequencies and heatmap generated by TBtools (Chen et al., 2020). Chromosome pairing was analyzed at metaphase I, and a quantitative cytological method was used to analyze the number of chiasmata (Moran et al., 2001). One-way analysis of variance (ANOVA) was performed using Origin Pro 2019.^1^ ANOVA was performed on the number of chiasmata of the three subgenomes and each chromosome within the subgenomes.

## Results

### Molecular Karyotype of the Investigated Sample

**FIGURE 1:**
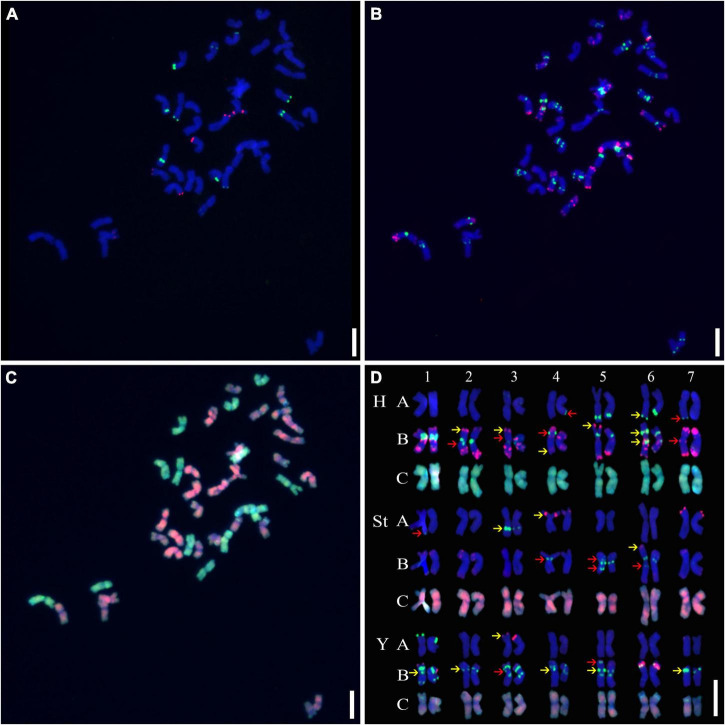
Sequential fluorescence *in situ* hybridization (FISH) on mitotic chromosomes and karyotypes of plant A02-1 with repetitive sequence probes. **(A)** pTa71-2 (red) and pSc119.2 (green); **(B)** pAs1 (red) and (AAG)_10_ (green); **(C)** genomic DNA probes of *P. stipifolia* (red); and *H. bogdanii* (green). **(D)** The molecular karyotype of A02-1 of *E. nutans*. Red arrows: the presence or absence of FISH signals between homologous chromosomes; yellow arrows: hybridization intensity discrepancies of FISH signals between homologous chromosomes. Bars = 10 μm.

Three rounds of hybridization were successively conducted on the metaphase chromosomes of mitotic cells, and a detailed molecular karyotype was proposed, with each chromosome distinctly distinguished (Figures 1A–C). Chromosome heterozygosity was measured by polymorphisms in the presence/absence or distinct hybridization intensity discrepancies of the tandem repeats used between homologous chromosomes. This showed that many chromosomes in A1-01 were in a heterozygous state. Except for 1H, 2St, 7St, and 6Y, distinct polymorphisms were detected in all chromosomes (Figure 1D).

### Abnormality Types in Different Meiosis Stages and Different Genomes

**FIGURE 2:**
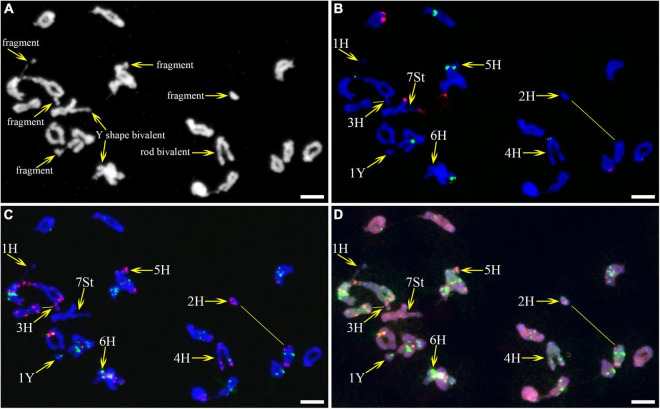
Fluorescence *in situ* hybridization (FISH) patterns at meiotic diakinesis I of plant A02-1. **(A)** Chromosomes stained with 4’,6-diamidino-2-phenylindole (DAPI); **(B)** pTa71-2 (red) and pSc119.2 (green); **(C)** pAs1 (red) and (AAG)_10_ (green); **(D)** genomic DNA probes of *P. stipifolia* (red) and *H. bogdanii* (green). Yellow arrows: Abnormal paired homologous chromosomes; Yellow lines: link chromosome fragments to their locations. Bars = 10 μm.

**FIGURE 3:**
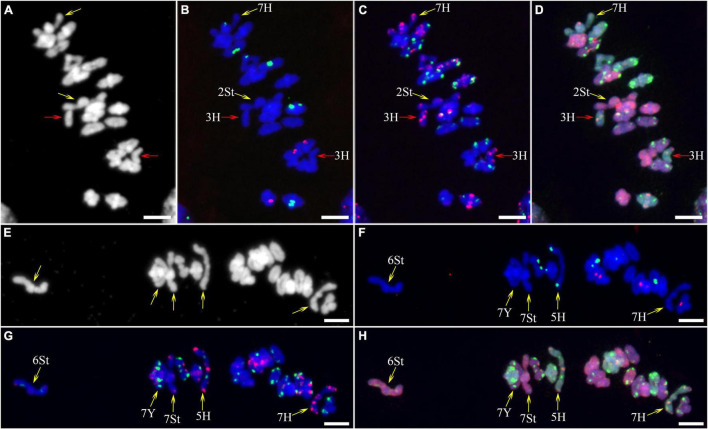
Fluorescence *in situ* hybridization (FISH) patterns at meiotic metaphase I of plant A02-1 of *E. nutans.* **(A,E)** Chromosomes stained with DAPI; **(B,F)** pTa71-2 (red) and pSc119.2 (green); **(C,G)** pAs1 (red) and (AAG)_10_ (green); **(D,H)** genomic DNA probes of *P. stipifolia* (red) and *H. bogdanii* (green). Yellow arrows: rod bivalents; red arrows: univalents. Bars = 10 μm.

**FIGURE 4:**
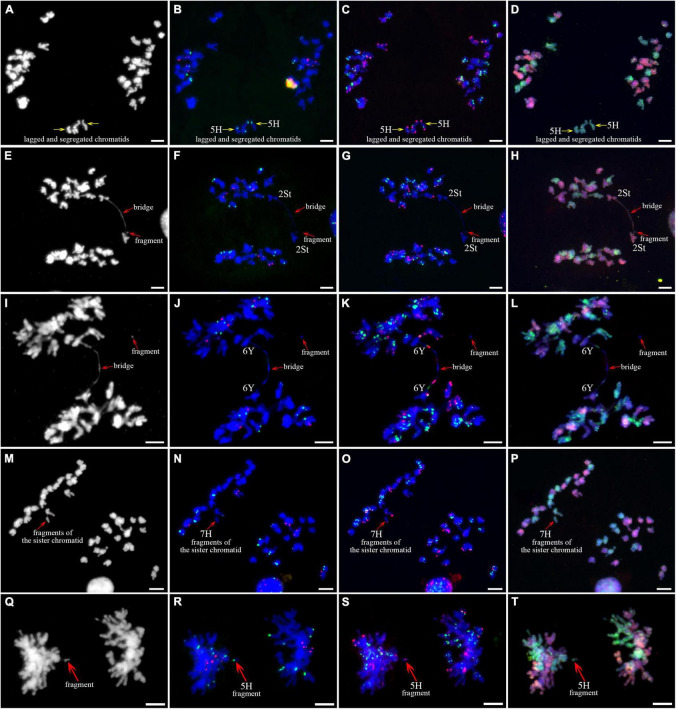
Sequential fluorescence *in situ* hybridization (FISH) of meiotic anaphase I of plant A02-1 of *E. nutans.* **(A,E,I,M,Q)** Chromosomes stained with DAPI; **(B,F,J,N,R)** pTa71-2 (red) and pSc119.2 (green); **(C,G,K,O,S)** pAs1 (red) and (AAG)_10_ (green); **(D,H,L,P,T)** genomic DNA probes of *P. stipifolia* (red) and *H. bogdanii* (green). Bars = 10 μm.

**FIGURE 5:**
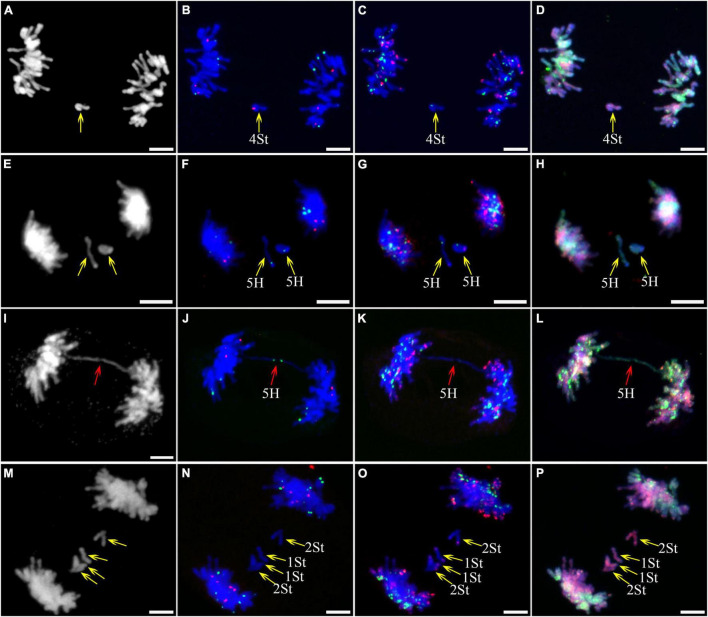
Sequential fluorescence *in situ* hybridization (FISH) of meiotic ana-telophase II of plant A02-1 of *E. nutans.* **(A,E,I,M)** Chromosomes stained with DAPI; **(B,F,J,N)** pTa71-2 (red) and pSc119.2 (green); **(C,G,K,O)** pAs1 (red) and (AAG)_10_ (green); **(D,H,L,P)** genomic DNA probes of *P. stipifolia* (red) and *H. bogdanii* (green). Yellow arrows: lagged chromosomes; red arrows: bridge. Bars = 10 μm.

As described in metaphase chromosomes in mitotic cells, each chromosome can also be identified by distinct FISH patterns by successive hybridization in a few meiosis stages. In this study, the cytogenetic investigation was carried out in the diakinesis (Figure 2), metaphase I (Figure 3), anaphase I (Figure 4), and ana-telophase II (Figure 5), where abnormal chromosomes can be more easily observed and identified than those in the other stages. Additionally, the uninucleate stage of the pollen was investigated (Supplementary Figure 1). A total of 3,477 cells were observed in the meiosis stages, of which the least number of cells were in the diakinesis stage (54) and the majority were in the uninucleate stage (2,604) (Supplementary Table 1). Different chromosomal abnormality types were revealed in different stages. In the diakinesis stage, three types of abnormalities—univalent, fragment, and rod/Y shape bivalent were revealed. Metaphase I showed two types of abnormalities—univalent and rod bivalent; anaphase I, six types—lagged and segregated chromatids, bridge, fragment of the sister chromatid, fragment, bridge accompanied with fragment, and unequal segregation of chromosomes; and ana-telophase II, two types—lagged chromatid and bridge.

**FIGURE 6:**
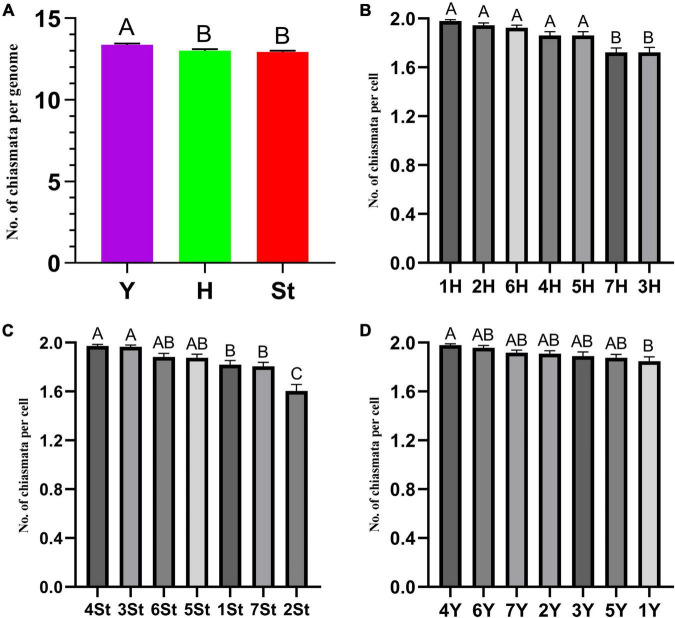
ANOVA of chromosome chiasmata number. **(A)** Between genomes; **(B–D)** within genomes. Experimental data are presented as the mean ± standard error of the mean. The different Roman letters in the superscript of each column indicates that the difference between the two means is very significant (*p* < 0.01).

The types and frequencies of abnormalities varied among the different genomes. Statistical investigation of chiasmata variance in each genome during metaphase I demonstrated that the chiasmata of the St and H genomes were distinctly lower than those of the Y genome (Supplementary Table 2 and Figure 6A). Unstable St genome chromosomes were observed in most stages (Supplementary Table 3). It showed the highest frequencies of univalent (0.529%) and rod/Y shape bivalent (6.878%) in diakinesis; the highest univalent (1.785%) in metaphase I; the highest bridge (0.314%) and bridge accompanied with fragment (0.314%) and unequal segregation of chromosomes (0.471%) in anaphase I; and the highest lagged chromatid in ana-telophase II (0.453%). Meanwhile, the St genome contained the highest frequency of micronuclei (2.611%) in the uninucleate stage. The Y genome chromosomes demonstrated the lowest frequencies of abnormalities in nearly all the investigated stages. The H genome showed the highest frequency of fragments (25.132%) in diakinesis; the highest rod bivalents (12.698%) in metaphase I; and frequencies of lagged and segregated chromatids (0.471%), lagged fragments (0.471%), fragments of the sister chromatids (0.471%) in anaphase I; and a high rate of bridges in ana-telophase II (0.342%) (Supplementary Table 3).

### Abnormalities in Individual Chromosomes

**FIGURE 7:**
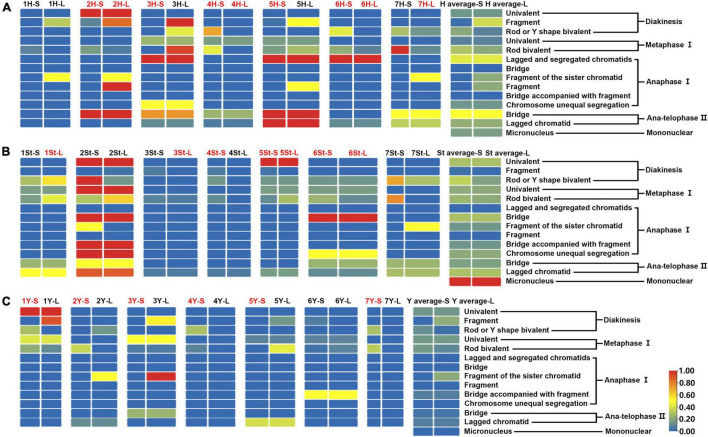
Heatmap generated by TBtools showing the types and frequencies of the abnormality chromosomes. The scale bars represent the relative frequencies of the abnormality chromosomes. The arms with the red name represent heterozygosity, and the black represents homozygosity. The complete heatmap is divided into three parts **(A–C)** by genome for demonstration purposes.

The types and frequencies of abnormalities vary between different chromosomes. Statistical investigation of the variance of chiasmata of each genome in metaphase I demonstrated that the chiasmata number of 3H and 7H were very significantly lower than that of other chromosomes in the H genome; in the St genome, the chiasmata number of 2St was very significantly lower than that of other chromosomes, and the chiasmata number of 4St and 3St was very significantly higher than 1St, 2St and 7St; the chiasmata number of 1Y was very significantly lower than that of 4Y in the Y genome (Supplementary Table 4 and Figures 6B–D). However, the frequencies of abnormalities in the different chromosomes varied at different stages (Supplementary Table 3). The chromosomal distribution of the types and frequencies of abnormalities is illustrated using a heatmap for convenient comparison (Figure 7).

**FIGURE 8:**
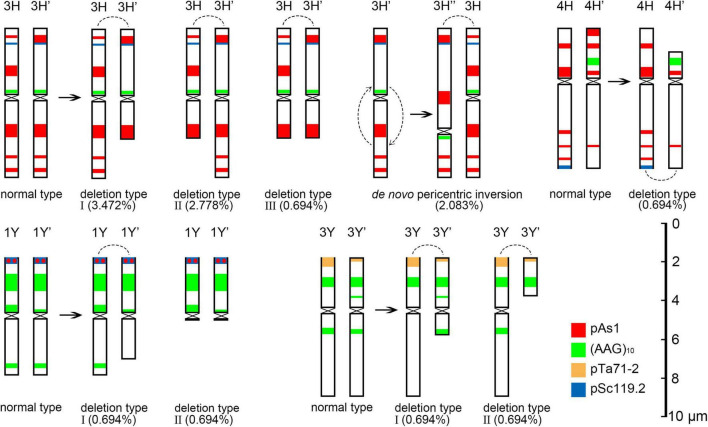
Schematic diagram of discernible chromosomal structural variation at metaphase I. The arc is the joint position in the rod bivalent.

The 3H chromosome was revealed to be the most unstable H genome chromosome, which covered the unstable types in nearly all meiosis stages, especially with the highest rates of fragments in diakinesis (62.963%) and univalents in metaphase I (2.083%) (Figure 7 and Supplementary Table 3). The high frequencies of 3H fragments in diakinesis reflect the possibility of chromosome deletion in subsequent development. A detailed comparison between diakinesis and metaphase I in 3H chromosomes revealed three types of deletions in terminal positions of the long arms in each homologous chromosome or both, with frequencies of 3.472, 2.778, and 0.694%, respectively. Specifically, a tentative *de novo* pericentric inversion in metaphase I in three of 144 cells (2.083%) was identified in 3H (Supplementary Figure 2A and Figure 8). One breakpoint of the inversion in the long arm responded somewhat to the breakpoints of these deletions, strongly suggesting that they may share a common DSBs site on the 3H chromosome. Comparison of each chromosome between the diakinesis stages and metaphase I revealed a low frequency (0.694%) of a terminal deletion in the short arm of 4H (Supplementary Figure 2B and Figure 8), which corresponds to the highest rate of short arm rods or Y shape bivalents in diakinesis. Lagged and segregated chromatids in anaphase I were observed in 3H, 5H, and 6H. The lagged fragments in anaphase I were detected in 2H and 5H, whereas fragments of the sister chromatid were identified on chromosomes 1H, 2H, and 7H in anaphase I (Figure 4 and Supplementary Table 3). Five of the seven H genome chromosomes displayed frequencies of bridges in ana-telophase II (Figure 5 and Supplementary Table 3). All H genome chromosomes with bridges in ana-telophase II had fragment or rod bivalent abnormality types in diakinesis. However, two chromosomes, 1H and 6H, with abnormality types of fragments or rod bivalents in diakinesis, did not show any bridges in ana-telophase II (Supplementary Table 3).

Chromosome 2St was the most unstable chromosome in the St genome, which showed the highest frequency of abnormality at nearly every stage (Figure 7 and Supplementary Table 3). In particular, the bridge accompanied by a fragment, with a frequency of 2.198%, was identified in anaphase I (Figures 4E–H). The bridge accompanied by a fragment in anaphase I and the bridge in ana-telophase II reflects the result of the typical meiotic crossover between homologous regions of an inversion (specialty of paracentric inversion) under heterozygous conditions. This suggests that chromosome 2St is involved in paracentric inversion. Low frequencies of fragments were observed in 2St, 3St, 4St, and 5St in diakinesis. Comparing each chromosome between the diakinesis stages and metaphase I identified no terminal deletions in all St genome chromosomes. Chromosome 6St was identified, showing low frequencies of lagged and segregated chromatids and bridges in anaphase I. A fragment of sister chromatids 7St was found at anaphase I. Three of the seven chromosomes (1St, 6St, and 7St) showing no fragments in diakinesis displayed bridges in ana-telophase II. All St chromosomes were detected to present the frequencies of lagged chromatids in ana-telophase II (Supplementary Table 3).

Most of the Y genome chromosomes were more stable than the St and H, especially in anaphase I and ana-telophase II (Figure 7). Chromosomes 1Y and 3Y showed higher frequencies of fragments in diakinesis and univalents in metaphase I than those in the others. Comparing each Y chromosome between the diakinesis stages and metaphase I revealed two types of deletions in 1Y and 3Y, respectively, with a low frequency of 0.694% for each type. One 1Y deletion type was identified as a break in the distal parts of the long arm, whereas the other was identified as a break near the centromere. One 3Y type was characterized as breaks in the interstitial parts of the long arm, while the other was identified as a break in the interstitial regions of the short arm (Figure 8 and Supplementary Figures 2C,D). Chromosome 2Y was detected at low frequencies in the fragment of the sister chromatid in anaphase I and laggard chromatid in ana-telophase II. Chromosome 6Y was identified as the low frequency of 1.099% bridges accompanied by fragments in anaphase I (Figures 4I–L). Chromosome 6Y tentatively involved paracentric inversion, as described in Section 2St. Because the abnormality frequency in anaphase I in 6Y was much lower than that in 2St, this indicates that 6Y may involve a much smaller inversion than 2St.

## Discussion

The correct completion of meiotic cell division involves a sequence of coordinated steps during the two phases of meiosis. In this study, chromosomal abnormalities were detected at nearly every division step. Homologous chromosome recognition is a key step during meiosis. The efficacy of meiosis relies heavily on the accuracy of the chromosome pairing. Chromosome pairing is more challenging in polyploid plants because of the presence of duplicated homologous or homoeologous chromosomes. In our study, a few chromosome pairing aberrations were observed; however, no homoeologous chromosome pairing between different genomes was detected. This suggests that homologous pairing in *E. nutans* is well regulated by genetic elements such as the *Ph1* locus in allopolyploid wheat (Riley and Chapman, 1958; Sears, 1976). The pairing of homologous chromosomes during meiosis is determined prior to and concomitant with meiotic recombination, referred to as recombination-independent and recombination-dependent chromosome pairing, respectively (Grusz et al., 2017). *Ph1* has been implicated in many recombination-independent events, such as centromere pairing preprophase (Martinez-Perez et al., 2001), presynaptic alignment of chromosomes (Feldman, 1993), and changes in chromosome condensation that prevent DSB repair by homologous chromosomes (Greer et al., 2012). In this study, chromosome pairing aberrations were detected in diakinesis and metaphase I on a few chromosomes, most of which also showed FISH pattern polymorphisms between homologs in mitotic cell chromosomes. This indicates that chromosome pairing aberration is leaded by the diverse sequences or structure of the homologous chromosomes, since the parents of the investigated hybrid may be differentiated in these chromosomes or chromosomal regions. The frequency of chromosome pairing varies between different chromosomes and genomes. The St and H genomes presented distinctly higher pairing aberrations than the Y genome. These results are in accordance with the cytological investigation of *E. nutans* with varying fertility, suggesting that the St and H genomes may contain more chromosomal structural variations than the Y genome (Liu et al., 2021).

Chromosomal fragments were observed in both diakinesis and anaphase I. The fragments in diakinesis were mostly identified as terminal fission, which was further confirmed by identifying deletions in the corresponding chromosomes during metaphase I. The pairing and subsequent segregation of homologous chromosomes are achieved by chromosomal recombination during meiosis I through the formation of DSBs (Zickler and Kleckner, 2015). DSBs are repaired by DSB repair or by non-homologous end-joining of DSBs (Szostak et al., 1983; Gaeta and Chris Pires, 2010). Errors during non-homologous end-joining of DSBs can result in insertions and deletions at the ligation site (Pipiras et al., 1998). Chromosome pairing in *E. nutans* is strictly regulated by the *Ph1* system, which prevents the repair of DSBs by homoeologous chromosomes (Greer et al., 2012). Therefore, the breaks in diakinesis in a few chromosomes that were not well paired were probably initiated by failing to repair the DSBs by non-homologous end-joining of DSBs. Two types of fragments were observed in anaphase I. The fragments in the equatorial plate were mostly derived from the crossover of homologous chromosomes involving paracentric inversions, which were confirmed by fragments along with bridges in a few cells. However, fragments were also detected in a few of poled chromosomes. A few of these were identified as chromatid breaks using distinct chromosomal markers. Sister chromatid cohesion is essential for faithful chromosome segregation, and the cohesion complex is highly conserved in eukaryotes (Zamariola et al., 2014). DSBs occurring in sister chromatids are repaired using the intact sisters as the repair template by homologous recombination through generating junction molecules. Defective resolution of junction molecules not only leaves DSBs unrepaired but also has the broken sisters remain entangled with the intact sisters, leading to the formation of isochromatid breaks, where both chromatids of a single chromosome are broken at the same sites, in mitotic chromosome spreads (Shimizu et al., 2020). The staggered breaks, either chromatid or isochromatid breaks, rather than ectopic exchange between inverted repetitive sequences, was suggested to be the prevalent mechanism for the generation of inversions in the *melanogaster* species group evidenced by genome sequences (Ranz et al., 2007). In this study, the chromosome fragments in diakinesis strongly suggested isochromatid breaks involvement. Especially, the identified *de novo* inversions in 3H was distinctly featured with isochromatid breaks. Whether the breaks formation in meiosis of *E. nutans* is the same as the chromatid breaks described in mitotic cells (Shimizu et al., 2020) needs further investigation. The sister chromatids with breaks were frequently heterozygous in anaphase I (Figures 4N,O), as are chromosomes with the isochromatid breaks in diakinesis (Figure 2). Most of the inversions identified in different ecotypes of sunflower are introgression chromosomal regions from related species (Todesco et al., 2020). If the inversions in sunflower are caused by isochromatids breaks, the sequences divergence of the introduced blocks should be considered to play a role to initiate the chromatid or isochromatid breaks. Given the high chromosome heterozygosity of the chromatid and isochromatid breaks in this study, the chromatids junction may be affected by structural variation or sequence divergence between them.

In this study, bridges were observed in anaphase I and anaphase II, whereas their frequencies were higher in anaphase II than in anaphase I. Breakage-fusion-bridge was classified as chromosome type and chromatid type by the breakage and fusion points between chromosomes and chromatids, respectively (Lukaszewski, 1995). Two main factors may be responsible for bridge formation in anaphase I. The bridges resulted from the crossover between homologous chromosomes involved in structural variations, such as inversion, and the others were *de novo* chromosome type breakage-fusion-bridge, derived from the breakage and fusion between homologous chromosomes in diakinesis. The frequency of fragments that occurred from chromosome breakage was higher in diakinesis than in bridges in anaphase I on a few chromosomes suggesting that the broken points were healed by initiated telomere sequences or that the breakages were fused between chromatids. The breakage and fusion in diakinesis between chromatids may be responsible for the high frequency of bridges in anaphase II.

Inversion, a kind of special chromosomal rearrangement, plays a very important role in speciation and local adaptation (Dobzhansky and Sturtevant, 1938; Rieseberg, 2001; Todesco et al., 2020). Inversions include pericentric inversion, which involves breakpoints at different distances from either side of the centromere, and paracentric inversion, where breakpoints occur on one arm at different distances from either side (Schubert, 2007). During meiosis, paracentric inversion distinctly features bridges and fragments during anaphase I. In this study, chromosome 2St showed a frequency of 2.198% bridges accompanied with fragments in anaphase I, which strongly suggests a paracentric inversion in 2St. Since more than one chromosome showed chromosome aberration in anaphase I and other steps, other pericentric inversions or paracentric inversions involving short inversed segments may still require further confirmation. In previous studies (Dou et al., 2009, 2017), many chromosome variations, except inversions, have been identified in mitotic cell analysis. In this study, inversion was first determined using meiosis analysis. *E. nutans* is widely distributed and adaptable to the Qinghai-Tibet Plateau. Whether the ecological differentiation of *E. nutans* is strongly associated with the variety of inversions still needs further elucidation. Chromosome inversion can be finely identified using advanced molecular cytogenetic methods capable of detecting structural variations using single-copy genes (Danilova et al., 2017; Said et al., 2018) and even using a new approach of long-read sequencing and optic mapping (Staňková et al., 2016; Ho et al., 2020). Our study showed that molecular cytogenetic investigation of meiosis also provided valuable information on chromosomal structural variation in a conventional and low-cost method, especially for polyploid Triticeae species with large genomes. In the future, more major inversions in *E. nutans* can be detected using a combination strategy.

*E. nutans* demonstrates a high frequency of variation of intra- and inter-population molecular karyotypes. The reason for this high chromosomal variation is unclear. In the present study, primary chromosomal structural variations derived from the misrepair of DSBs were exclusively identified, which was confirmed by the deletion of chromosomes and *de novo* inversions in metaphase I. In addition, secondary chromosomal structural variations resulting from the unstable products of primary structural variations were still uncovered. If the novel chromosome variants were successfully transferred to the next offspring from the gametes, the chromosome diversity would increase in this way in the populations. Meiosis abnormalities have also been reported in *E. nutans* plants from West Himalaya, India, depicting the phenomenon of cytomixis and associated meiotic abnormalities (viz. non-synchronous disjunction of bivalents, chromatin bridges, laggards, micronuclei, and shriveled microspores) (Singh et al., 2020). In particular, one of the three plants exhibited fragments in metaphase I, that were suggested to be B chromosomes (Singh et al., 2020). In our study, the fragments detected during diakinesis were exclusively identified as deleted fragments. Similar results have prompted the discovery of universal mechanisms responsible for meiosis abnormalities in *E. nutans* populations. Meiosis behavior of individual chromosomes was observed in the present study, and meiosis abnormality in the individuals was closely related to the heterozygous FISH patterns of the homologous chromosomes detected in mitotic cell metaphase. The polymorphism between homologous chromosomes suggests variation in chromosomal structure or sequence divergence in the parts or segments between them. Introgression hybridization with related species can introduce structural variations with divergent blocks in the genome (Todesco et al., 2020). *E. nutans* exhibits high cross-ability with other *Elymus* taxa (Lu, 1993), and interspecies hybrids have been frequently observed in the field (Lu et al., 2019). This suggests that the genome of *E. nutans* may contain diverged chromosomal segments due to introgression hybridization with related species. Meiotic recombination occurs predominantly among allelic regions of homologous chromosomes (Naranjo and Corredor, 2008). In the heterozygous state, the introduced homeologous or non-homologous segment from the same genome of the related species may be responsible for the recombination failing between the homologous chromosomes and triggers the meiosis abnormalities. Transposable elements can cause chromosomal breakage and promote chromosomal aberrations, such as translocations, inversions, deletions, duplications, and fragment formation (McClintock, 1946; Kunze et al., 1997; Bennetzen, 2000; Kidwell and Holyoake, 2001). Bursts in transposable elements can be driven by stress, environmental change, and interspecific hybridization (Belyayev, 2014). Whether activated transposable elements in a heterozygous state induce meiosis chromosomal abnormalities requires further investigation. Chromosomal variation plays an important role in the high genetic diversity and wide adaptation of *E. nutans*, although the mechanism to maintain it is intriguing. Investigating diverse *E. nutans* plants widely in a natural state or under controlled conditions using a combination of cytological and genomic tools may elucidate this phenomenon in the future.

## Data Availability Statement

The original contributions presented in the study are included in the article/Supplementary Material, further inquiries can be directed to the corresponding author.

## Author Contributions

QD conceived and designed the experiments. BL performed the experimental work. QD and BL analyzed the data and wrote the manuscript. XT assisted in experimental work. All authors contributed to the article and approved the submitted version.

## Conflict of Interest

The authors declare that the research was conducted in the absence of any commercial or financial relationships that could be construed as a potential conflict of interest.

## Publisher’s Note

All claims expressed in this article are solely those of the authors and do not necessarily represent those of their affiliated organizations, or those of the publisher, the editors and the reviewers. Any product that may be evaluated in this article, or claim that may be made by its manufacturer, is not guaranteed or endorsed by the publisher.

## Footnotes

1
https://www.originlab.com/2019
