# Supplementary material for: Meiotic Chromosomal Abnormality Detected in a Heterozygote of Elymus nutans
Source: Front Plant Sci. 2022 May 3;13:895437. doi: 10.3389/fpls.2022.895437 (PMC9112040; doi:10.3389/fpls.2022.895437)
Supplement: Supplementary file 2 [file Data_Sheet_1.docx]

Supplementary Material

# Supplementary Figures


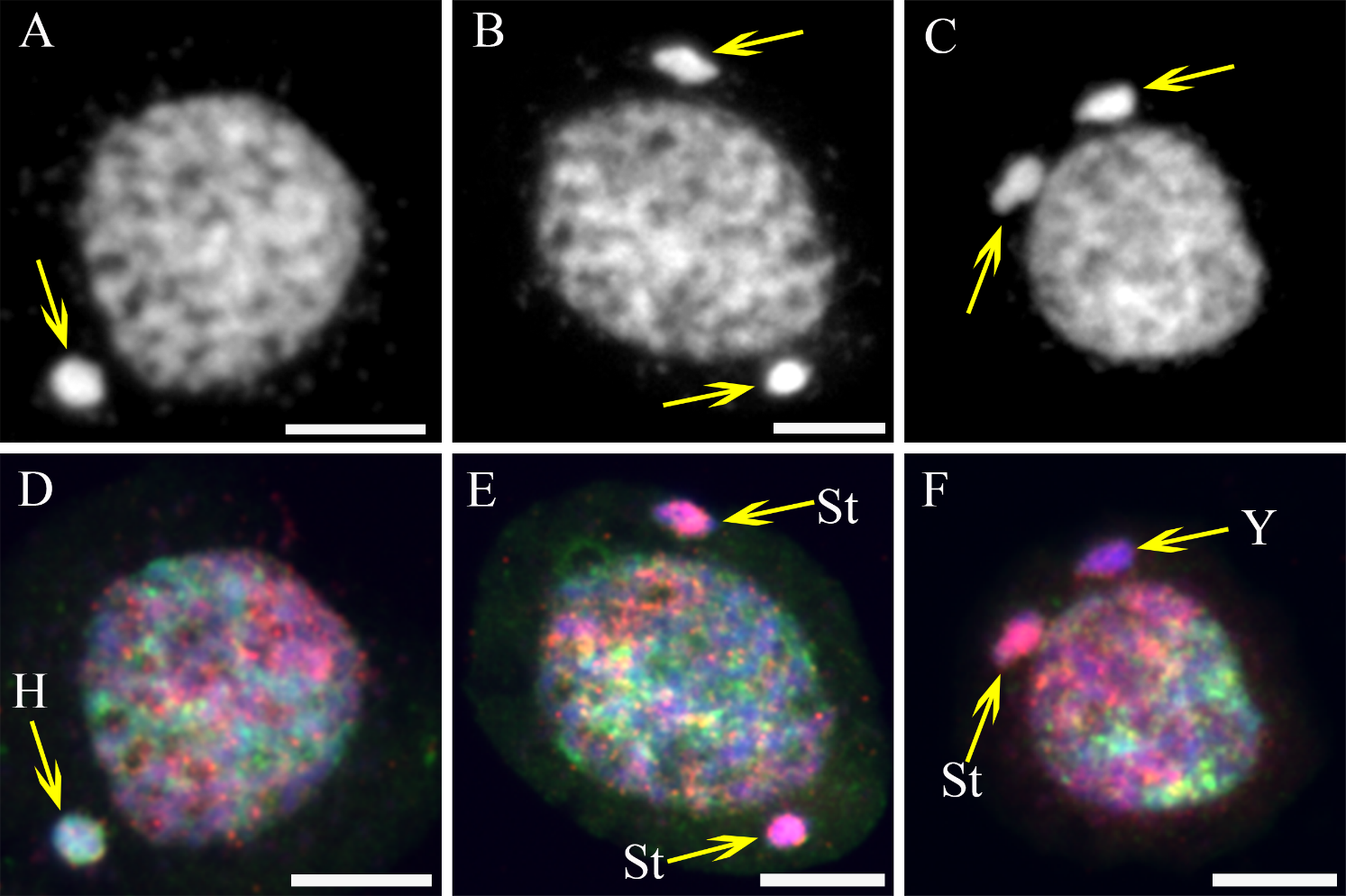


**Supplementary Figure 1.** Micronucleus at uninucleate stage. (A-C) Chromosomes stained with DAPI; (D-F) genomic DNA probes of *P. stipifolia* (red) and *H. bogdanii* (green). Yellow arrows: micronucleus. Bars = 10 μm.


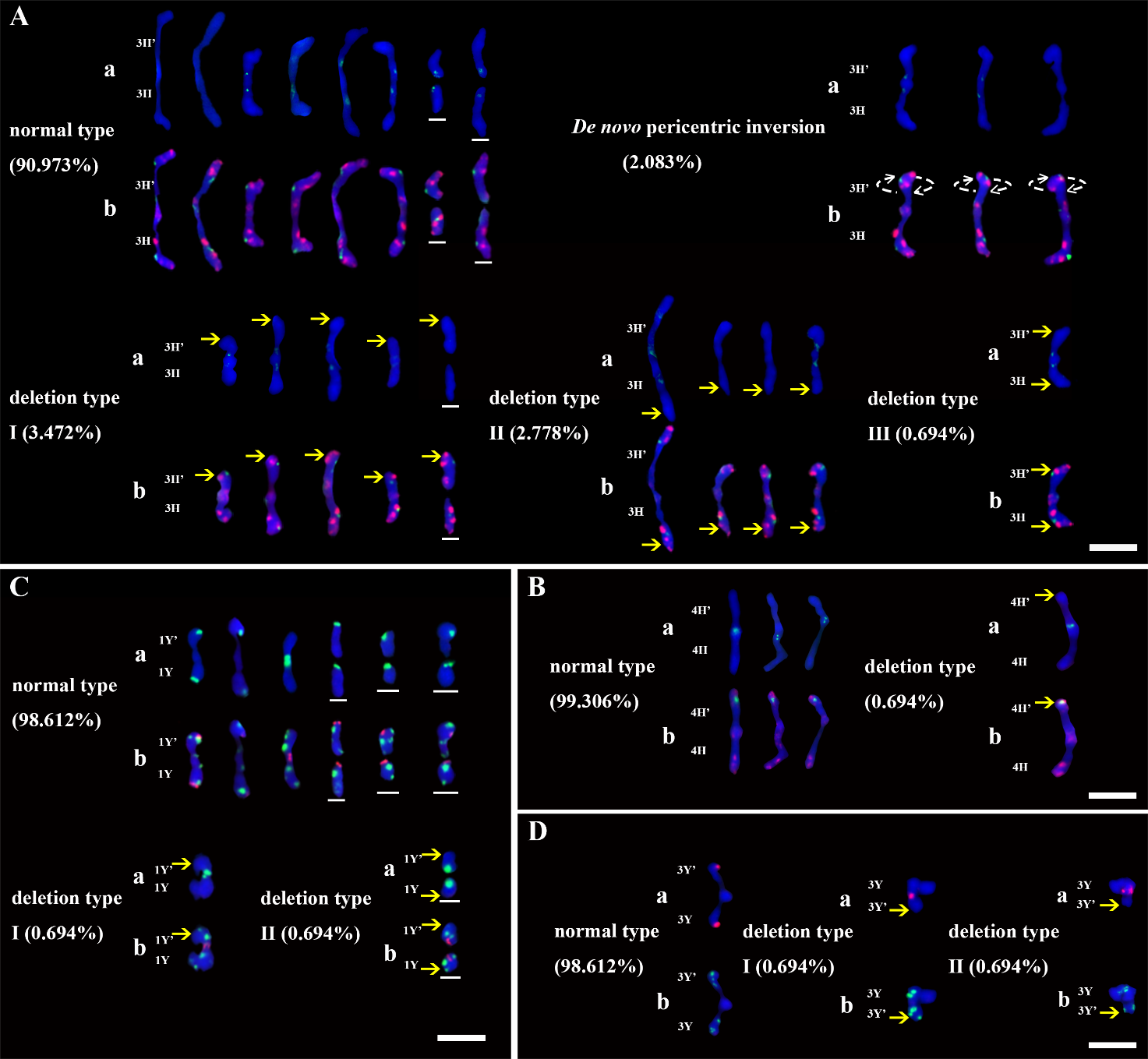


**Supplementary Figure 2.** Discernible chromosomal structural variation at rod bivalents and univalents from metaphase Ⅰ. All rod bivalents and univalents with chromosomal structural variations are shown, while only a portion of rod bivalents and univalents without chromosomal structural variation are shown as controls (normal type). In order to facilitate the observation of chromosomal structural variation sites in univalents, we arranged a few of univalents as rod bivalents forms (with white lines below). Chromosome structure was determined simultaneously by using two probe combinations (a and b) arranged upper and below respectively in each plate. a: pTa71-2 (red) and pSc119.2 (green); b：pAs1 (red) and (AAG)_10_ (green). A pair of homologous chromosomes with polymorphic signal are distinguished by the presence or absence of “ ’ ”. A: *De novo* pericentric inversions; deletion type Ⅰ (deletion of pAs1 in 3H’ terminal); deletion type Ⅱ (deletion of pAs1 in 3H terminal); deletion type Ⅲ (deletion of pAs1 in 3H and 3H’ terminal) in chromosomes 3H; B: deletion type in 4H (deletion of pAs1 in 4H’ terminal ); C: deletion type Ⅰ (deletion of (AAG)_10_ in 1Y’ terminal) and deletion type Ⅱ (deletion of long arms in 1Y and 1Y’) identified in 1Y; D: deletion type Ⅰ (deletion part of long arm in 3Y’) and deletion type Ⅱ (deletion long and part of the short arm in 3Y’) identified in 3Y. Yellow arrow: deletion position. Bars = 10 μm.

# Supplementary Tables

**Supplementary Table 1.** The number of cells investigated at each meiosis stage in A02-1.

| **Meiosis stage** | **No. of cells** |
| --- | --- |
| Diakinesis | 54 |
| Metaphase Ⅰ | 144 |
| Anaphase Ⅰ | 91 |
| Ana-Telophase Ⅱ | 584 |
| Mononuclear | 2604 |
| Total | 3477 |

**Supplementary Table 2.** ANOVA of the number of chiasmata in each genome.

| **Genome** | **Mean** |
| --- | --- |
| Y | 13.375 A |
| H | 13.014 B |
| St | 12.924 B |

**Note:** The different Roman letters of each column indicates that the difference between the two means is very significant (*p* < 0.01).

**Supplementary Table 4.** ANOVA of the number of chiasmata in each chromosome.

| **Chromosome** | **Mean** | **Chromosome** | **Mean** | **Chromosome** | **Mean** |
| --- | --- | --- | --- | --- | --- |
| 1H | 1.979 A | 4St | 1.972 A | 4Y | 1.979 A |
| 2H | 1.944 A | 3St | 1.965 A | 6Y | 1.958 AB |
| 6H | 1.924 A | 6St | 1.882 AB | 7Y | 1.917 AB |
| 4H | 1.861 A | 5St | 1.875 AB | 2Y | 1.910 AB |
| 5H | 1.861 A | 1St | 1.819 B | 3Y | 1.889 AB |
| 7H | 1.722 B | 7St | 1.806 B | 5Y | 1.875 AB |
| 3H | 1.722 B | 2St | 1.604 C | 1Y | 1.847 B |

**Note:** The different Roman letters of each column indicates that the difference between the two means is very significant (*p* < 0.01).
